# Development and validation of an artificial neural network prognostic model after gastrectomy for gastric carcinoma: An international multicenter cohort study

**DOI:** 10.1002/cam4.3245

**Published:** 2020-07-15

**Authors:** Ziyu Li, Xiaolong Wu, Xiangyu Gao, Fei Shan, Xiangji Ying, Yan Zhang, Jiafu Ji

**Affiliations:** ^1^ Gastrointestinal Cancer Center Key Laboratory of Carcinogenesis and Translational Research (Ministry of Education) Peking University Cancer Hospital and Institute Beijing China

**Keywords:** artificial neural network, gastric cancer, prediction, survival

## Abstract

**Background:**

Recently, artificial neural network (ANN) methods have also been adopted to deal with the complex multidimensional nonlinear relationship between clinicopathologic variables and survival for patients with gastric cancer. Using a multinational cohort, this study aimed to develop and validate an ANN‐based survival prediction model for patients with gastric cancer.

**Methods:**

Patients with gastric cancer who underwent gastrectomy in a Chinese center, a Japanese center, and recorded in the Surveillance, Epidemiology, and End Results database, respectively, were included in this study. Multilayer perceptron neural network was used to develop the prediction model. Time‐dependent receiver operating characteristic (ROC) curves, area under the curves (AUCs), and decision curve analysis (DCA) were used to compare the ANN model with previous prediction models.

**Results:**

An ANN model with nine input nodes, nine hidden nodes, and two output nodes was constructed. These three cohort's data showed that the AUC of the model was 0.795, 0.836, and 0.850 for 5‐year survival prediction, respectively. In the calibration curve analysis, the ANN‐predicted survival had a high consistency with the actual survival. Comparison of the DCA and time‐dependent ROC between the ANN model and previous prediction models showed that the ANN model had good and stable prediction capability compared to the previous models in all cohorts.

**Conclusions:**

The ANN model has significantly better discriminative capability and allows an individualized survival prediction. This model has good versatility in Eastern and Western data and has high clinical application value.

## Graphical abstract

Using a multinational cohort, this study developed and externally validated an artificial neural network‐based survival prediction model for patients with gastric cancer after gastrectomy.

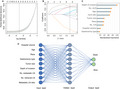

## 1 INTRODUCTION

Gastric cancer is the sixth most frequently diagnosed cancer and the second leading cause of cancer‐related death worldwide.^1^ Surgical resection is still the mainstay treatment for resectable gastric cancer; however, even after a potentially curative resection, a large proportion of patients develop tumor recurrence.^2^ Thus, survival prediction models are needed for planning additional treatments and an appropriate follow‐up schedule.

Currently, gastric cancer staging is according to the American Joint Committee on Cancer (AJCC) tumor‐node‐metastasis (TNM) staging system. However, due to the need for simplicity and uniform application, the TNM staging system does not account for many essential factors that can significantly affect patient survival in gastric cancer, including age, tumor location, histology, and ratio of positive to retrieved lymph nodes; thus, survival markedly varies for tumors of the same stage.^3^, ^4^, ^5^, ^6^, ^7^, ^8^

Various nomograms and scoring systems have been developed to achieve a superior method for predicting patient survival.^6^, ^7^, ^9^, ^10^ Some of these have been externally validated with high predictive value.^11^, ^12^, ^13^, ^14^ However, most models have been developed at specialized institutions and may not perform as well in the general population.^15^, ^16^, ^17^, ^18^ Thus, due to the lack of generalizability, none of the models have been widely used in the clinical practice by far.^19^

The occurrence and development of gastric cancer is very complicated, and many clinicopathological features can affect the prognosis, and most of them exhibit a multidimensional and nonlinear relationship. The artificial neural network (ANN) is a novel computer model and is especially suitable for dealing with nonlinear problems and analyzing multidimensional databases. ANN has already been applied successfully in the clinical setting for diagnosis and outcome prediction of multiple diseases.^20^, ^21^, ^22^, ^23^, ^24^, ^25^, ^26^ Promising results for survival prediction of gastric cancer have also been reported.^27^, ^28^, ^29^, ^30^, ^31^ However, a common problem in these studies is the lack of external validation and the small sample size, which may result in insufficient ANN training.

This study aimed to develop and validate a new survival prediction model for gastric cancer using an ANN. To improve the generalizability of our model, we used data from the Surveillance, Epidemiology, and End Results (SEER) for model development. Furthermore, two patient cohorts from China and Japan were also included for analysis.

## 2 METHODS

### 2.1 SEER cohort

Population‐based gastric cancer data were obtained from the SEER 18 registries, of which the newest database, “Incidence‐SEER 18 Regs Custom Data (with additional treatment fields), Nov 2018 Sub (1975‐2016 varying)” was reviewed. Gastric cancer patients were included using the following inclusion criteria: (a) single primary microscopically confirmed stomach cancer (ICD‐0‐3 site codes, C16.0 to C16.9), (b) histology codes of adenocarcinoma (ICD‐0‐3 histology/behavior codes, M‐8140/3, M‐8142/3 to M‐8145/3, M‐8210/3, M‐8211/3, M‐8255/3, M‐8260/3 to M‐8263/3, M‐8310/3, M‐8323/3, M‐8480/3, M‐8481/3, M‐8490/3), (c) curative intent gastrectomy with detailed surgery type according to the Surg Prim Site (1998+), and (d) no distant metastasis. Adjuvant chemoradiotherapy (CRT) has become the recommended treatment for gastric cancer in the United States based on the INT0116 clinical trial showing that adjuvant CRT yields better overall survival (OS) than surgery alone in 2001.^32^ Accordingly, we only included patients who were diagnosed between 2002 and 2015 to avoid differences in survival rates before 2001 and after 2001. Finally, 11 006 patients with detailed clinicopathologic information were included (Figure S1).

### 2.2 Japanese and Chinese cohorts

Our study used a dataset from a Japanese institute, Cancer Institute Ariake Hospital (CIAH), which maintains a large prospective gastric cancer database available for open use for academic purposes.^33^ Because the recording code is different from the SEER database, the inclusion criteria of the cohort are as follows: (a) single primary gastric cancer with R0 resection, (b) adenocarcinoma (papillary, tubular, poorly differentiated, mucinous, and signet‐ring cell carcinoma), (c) no distant metastasis. After excluding patients with incomplete clinicopathological information, 3521 patients diagnosed between 1990 and 2007 were included (Figure S2). The Chinese cohort was recruited from the Gastrointestinal Cancer Center of Peking University Cancer Hospital & Institute (PUCHI). Between 1 January 2007 and 31 December 2015, we collected data of 1432 patients who satisfied the aforementioned inclusion criteria for the CIAH cohort (Figure S3).

### 2.3 Clinicopathological data

For all the cohorts, collected data included patient demographics (race, age, and sex), pathological characteristics (tumor location, size, differentiation, histology type, depth of invasion, number of retrieved lymph nodes [rLN], and number of metastatic lymph nodes [mLN]), and follow‐up data (survival time and vital status). Race was categorized as non‐Latino White, Latino White, Black, Korean, Japanese, Chinese, Others (including American Indian, Alaskan Native, Pacific Islander, and other Asian). Age was divided into four groups: <41, 41‐60, 61‐70, and > 70 years. Tumor location was categorized as proximal third (including gastroesophageal junction), middle and distal third, overlapping lesion, and stomach, not otherwise specified (C16.5, C16.6, and C16.9 in SEER cohort). Histology type was categorized as general adenocarcinoma, mucinous adenocarcinoma, and signet‐ring cell carcinoma; well‐differentiated and moderately differentiated types were classified as the differentiated group, while poorly differentiated, mucinous, and signet‐ring cell types were classified as the undifferentiated group based on the Japanese classification of gastric cancer.^34^ Depth of invasion and lymph node metastasis were categorized based on the 8th edition of the AJCC TNM staging system. The surgical procedures were summarized as partial gastrectomy and total gastrectomy. Additionally, we calculated the metastatic lymph nodes ratio (MLR) by dividing the number of mLN by the number of rLN. For more than 95% of patients with gastric cancer in the United States were treated in hospitals with less than 200 cases per year, we defined the SEER cohort to be from low‐volume centers.^35^ Meanwhile, CIAH and PUCHI were both among the largest Asian cancer centers, and thus these cohorts were regarded to be from high‐volume centers. There was no information on Lauren type in the SEER and CIAH cohort; as such, we only referred to a previous study ^36^ to define Lauren subtypes based on the ICD codes. This study was performed according to the Helsinki Declaration of 1964 and later versions, and was approved by the Ethics Committee of Peking University Cancer Hospital. Patients from the PUCHI cohort provided written informed consent.

### 2.4 ANN model development

The SEER cohort was from 18 registries; of these, we randomly selected four registries as a Western validation cohort. We merged the remaining 14 registries of the SEER cohort and the CIAH cohort as a model development cohort. The development cohort was randomly divided into a training cohort (70%) and a testing cohort (30%). The PUCHI cohort was defined as an Eastern validation cohort. The ANN applied in this study was a multilayer perceptron neural network, which was constructed to predict 5‐year survival status. Because no perfect method exists for designing an ideal ANN, we identified the optimal structure of input layer parameters by using a penalized Cox regression model with least absolute shrinkage and selection operator (LASSO) penalty based on the 1‐SE criteria. We used IBM‐SPSS software to train an ANN model. Training and cross validation were performed with 70% of the training cohort for training and 30% of the training cohort for testing assigned randomly to prevent overfitting. The number of nodes in the hidden layer ranged from 1 to 50. We set the type of training as batch, optimization algorithm as scaled conjugate gradient, initial Lambda as 0.0000005, initial Sigma as 0.00005, interval center as 0, and interval offset as 0.5. The hyperbolic tangent function was used as the activation function in the hidden layer. In addition, to output 5‐year survival probability, softmax function was used as the activation function in the output layer. The ANN training stopped when the maximum steps without a decrease in error was 1. Default options were used for other options.

### 2.5 Statistical analysis

Clinicopathologic variables were compared using Chi‐squared test or Fisher exact test for categorical variables and the Student *t* test or analysis of variance for continuous variables. The tumor size, number of rLN, and MLR were grouped using X‐tile software (Figure S4).^37^ The Kaplan‐Meier method was used for analysis of OS, and log‐rank test was used for comparison of survival among different groups. Time‐dependent ROC curve and area under curve (AUC) were compared to evaluate the performance of the prediction models. Decision curve analysis (DCA) was performed to determine the practical clinical value of the prediction models by quantifying the net benefits according to the threshold probabilities. All statistical analyses were conducted using SPSS version 19.0 and R software 3.6.0. Statistical significance was set at two‐sided *P* < .05.

## 3 RESULTS

### 3.1 Clinicopathologic characteristics of the three cohorts

All patient and tumor characteristics differed significantly between the three cohorts (Table 1). The mean age was 66.9 ± 13.6, 60.3 ± 11.2, 61.7 ± 11.7 years in the SEER, CIAH, and PUCHI cohorts, respectively. In the SEER cohort, non‐Latino White patients accounted for the highest proportion, at 38.1%, and the Eastern‐Asian patients including Chinese, Japanese, and Korean were about 16.7%. In the PUCHI cohort, more patients (36.4%) have undergone total gastrectomy than the other two cohorts. In the CIAH cohort, the proportion of early gastric cancer was relative higher as indicated by 63.5% of tumor extensions confined to the mucosa or submucosa. More than 15 lymph nodes were examined in approximately 94.6% and 94.1% of patients in the CIAH and PUCHI cohorts, respectively.

**TABLE 1:** Demographic and clinicopathologic variables

Variable	SEER (n = 11 006)		CIAH (n = 3521)		PUCHI (n = 1432)		*P* Value
	n	%	n	%	n	%	
Mean age (y)	66.9 ± 13.6		60.3 ± 11.2		61.7 ± 11.7		<.001
Sex							<.001
Male	6353	57.7	2371	67.3	997	69.6	
Female	4653	42.3	1150	32.7	435	30.4	
Race							
Non‐Latino White	4188	38.1					
Latino‐White	2265	20.6					
Black	1700	15.4					
Chinese	591	5.4			1432	100	
Japanese	472	4.3	3521	100			
Korean	765	7					
Others	1025	9.3					
Tumor location							<.001
Upper	1380	12.6	733	20.8	311	21.7	
Middle	1233	11.2	1710	48.6	294	20.5	
Lower	4397	40	979	27.8	761	53.1	
Overlapping lesion	859	7.8	99	2.8	66	4.6	
Unknown	3137	28.5					
Tumor size (cm)							<.001
<2.6	2692	24.5	1231	35	478	33.4	
2.6‐6.6	5126	46.6	1730	49.1	749	52.3	
>6.6	2010	18.3	515	14.6	188	13.1	
Diffuse	219	2	45	1.3	17	1.2	
Unknown	959	8.7					
Gastrectomy type							<.001
Subtotal	8530	77.5	2790	79.2	911	63.6	
Total	2476	22.5	731	20.8	521	36.4	
Tumor extension							<.001
Mucosa	987	9	1281	36.4	197	13.8	
Submucosa	1688	15.3	954	27.1	158	11	
Proper muscle	1516	13.8	395	11.2	222	15.5	
Subserosa	3973	36.1	417	11.8	313	21.9	
Serosa	2329	21.2	433	12.3	503	35.1	
Adjacent organ invasion	513	4.7	41	1.2	39	2.7	
No. of rLN							<.001
1‐15	6034	54.8	192	5.5	84	5.9	
16‐29	3575	32.5	946	26.9	639	44.6	
>29	1397	12.7	2383	67.7	709	49.5	
No. of mLN							<.001
0	4723	42.9	2499	71	604	42.2	
1‐2	1982	18	466	13.2	239	16.7	
3‐6	1940	17.6	346	9.8	218	15.2	
7‐15	1697	15.4	178	5.1	245	17.1	
>15	664	6	32	0.9	126	8.8	
MLR							<.001
0	4723	42.9	2499	71	604	42.2	
<0.32	2842	25.8	979	27.8	583	40.7	
0.32‐0.64	1735	15.8	40	1.1	189	13.2	
>0.64	1706	15.5	3	0.1	56	3.9	
Differentiation							<.001
Differentiated	3474	31.6	1657	47.1	749	52.3	
Undifferentiated	7532	68.4	1864	52.9	683	47.7	
Histology							<.001
Adenocarcinoma	8341	75.8	2502	71.1	1028	71.8	
Mucinous Adenocarcinoma	263	2.4	59	1.7	71	5	
Signet‐ring cell carcinoma	2402	21.8	960	27.3	333	23.3	
Lauren type							<.001
Intestinal	2974	27	1716	48.7	323	22.6	
Diffuse	3231	29.4	960	27.3	367	25.6	
Mixed					742	51.8	
Unspecified	4801	43.6	845	24			
TNM 8th stage							<.001
IA	760	6.9	1862	52.9	263	18.4	
IB	432	3.9	391	11.1	143	10	
IIA	713	6.5	276	7.8	132	9.2	
IIB	597	5.4	244	6.9	191	13.3	
IIIA	825	7.5	348	9.9	251	17.5	
IIIB	953	8.7	173	4.9	239	16.7	
IIIC	692	6.3	35	1	129	9	
Cannot be staged	6034	54.8	192	5.5	84	5.9	
Median follow‐up time (month)	29		61		51		

Abbreviations: CIAH, Cancer Institute Ariake Hospital; mLN, metastatic lymph nodes; MLR, metastatic lymph nodes ratio; PUCHI, Peking University Cancer Hospital & Institute; rLN, retrieved lymph nodes; SEER, Surveillance, Epidemiology, and End Results.

### 3.2 Optimal variables selection and prediction model construction

**FIGURE 1:**
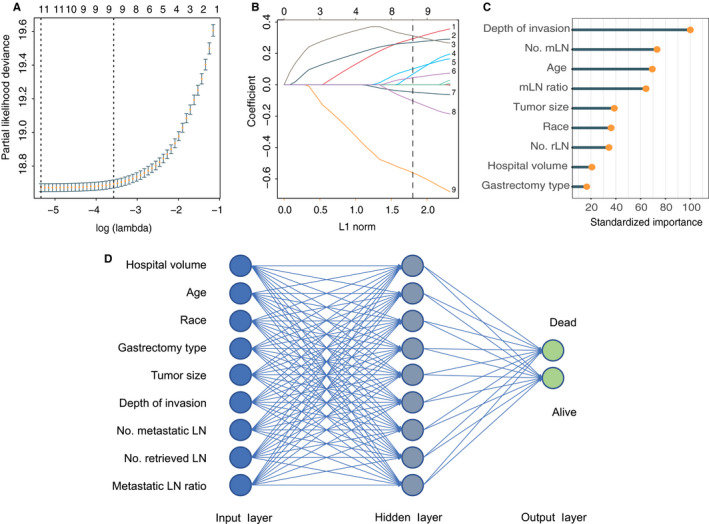
ANN model construction. A, Partial likelihood deviance for the LASSO coefficient profiles. The vertical dotted lines represent values according to the minimum (left line) and the 1‐SE (right line) criteria with fivefold cross‐validation. B, LASSO coefficient profiles of the nine selected clinicopathologic variables. The vertical dotted line is drawn corresponding to the optimal value. C, Independent standardized importance of selected variables in the trained ANN model. D, The framework of the ANN model including one input layer with nine nodes, one hidden layer with nine nodes, and one output layer with two nodes

The original three cohorts were processed as described above (Figure S5). The development cohort comprising 12 108 patients was randomly divided into a training cohort with 8475 patients and a testing cohort with 3633 patients (Table S1). Using the LASSO Cox regression model, we identified an optimal combination of parameters for predicting survival based on the training cohort (Figure 1A). This combination contains the following nine parameters: hospital volume, age, race, gastrectomy type, tumor size, depth of invasion, number of mLN, number of rLN, and MLR (Figure 1B). Then, we used these nine parameters to train an ANN model. In the end, the ANN model was constructed with a three‐layer neural network including nine input nodes, nine hidden nodes, and two output nodes (Figure 1D). The importance of the nine variables was standardized (Figure 1C). The most and least important variables were depth of invasion with 100% importance and the gastrectomy type with 16.6% importance, respectively. We exported an XML format file containing the trained model based on a standard of predictive model markup language (PMML; Supporting information). When validating the model externally, our defined parameter names and codes should be referred (Table S2).

### 3.3 Evaluation of the prediction ability of the ANN model

**FIGURE 2:**
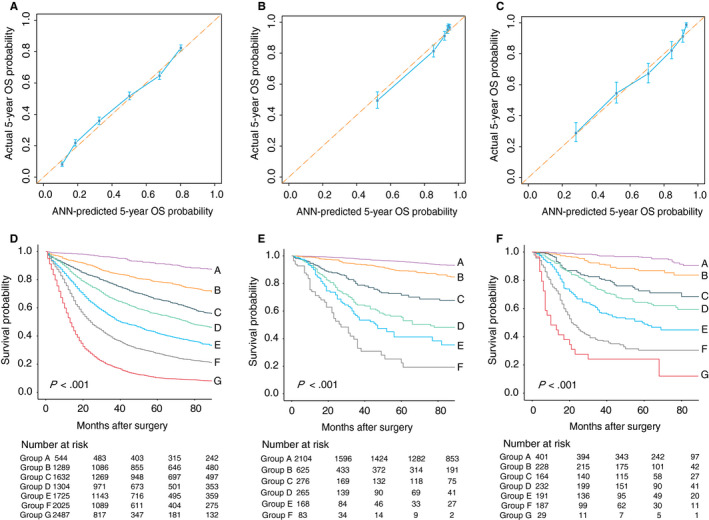
A‐C, Calibration of the ANN model in the SEER, CIAH, and PUCHI cohorts. The *x*‐axis and *y*‐axis represents the 5‐year survival probabilities predicted using the ANN model and the actual 5‐year survival rates, with 95% confidential interval, respectively. D‐F, Kaplan‐Meier survival curves of the ANN risk subgroups in the SEER, CIAH, and PUCHI cohorts

The predictive capability for survival of the ANN model and the AJCC TNM staging 8th edition was compared using time‐dependent ROC. In the Western validation cohort comprising 2419 patients, the predictive capability of ANN model for 5‐year survival was superior to that of the AJCC TNM staging 8th edition (AUC^ANN^, 0.789; 95% CI, 0.768‐0.809 vs. AUC^AJCC^, 0.738; 95% CI, 0.715‐0.760; *P* < .001). In the PUCHI validation cohort, the ANN model was also the better one (AUC^ANN^, 0.850; 95% CI, 0.826‐0.874 vs. AUC^AJCC^, 0.821; 95% CI, 0.795‐0.847; *P* < .001). Calibration plots for the predicted (ANN model) and actual 5‐year survival probability were created and showed good correspondence for all the cohorts (Figure 2A‐C, and Figure S6). The patients were divided into seven risk groups based on the survival probability predicted by the ANN model (equally divided from 0 to 1: group A with more than 0.857; group B, 0.714‐0.857; group C, 0.571‐0.714; group D, 0.429‐0.571; group E, 0.286‐0.429; group F, 0.143‐0.286; group G, less than 0.143). Kaplan‐Meier survival analysis was performed in the seven groups, and the survival curves are shown in Figure 2D‐F and Figure S7).

### 3.4 Comparison between the ANN model and previous prognostic models

**FIGURE 3:**
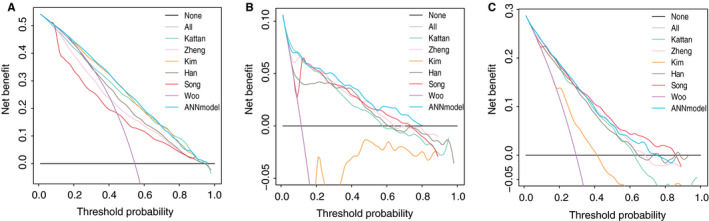
Decision curve analysis (DCA) of the 5‐year overall survival. A, SEER cohort. B, CIAH cohort. C, PUCHI cohort. The *y*‐axis represents the net benefit

After reviewing previous published articles, five nomograms and one scoring model were identified as available for all the three cohorts.^9^, ^10^, ^17^, ^38^, ^39^, ^40^ Referring to the involved variables for these six models, comparisons among the six models and our ANN model were performed in 3613 patients of the SEER cohort, 2676 patients of the CIAH cohort, and all patients of the PUCHI cohort. Both models of Kattan et al^10^ and Kim et al^38^ were developed from Western centers; in the SEER cohort, the AUC of these two models were not significantly different with that of our ANN model (Table 2). However, our ANN model was superior to the models developed from Eastern centers including the model by Zheng et al^39^ from China and the models by Han et al,^40^ Song et al,^17^ and Woo et al^9^ from Korea. In the CIAH cohort, the Western models did not perform as well as our ANN model. Furthermore, in the PUCHI cohort, our ANN model showed the best predictive capability. In the DCA, the ANN model showed better clinical usefulness than the other models in the SEER cohort (Figure 3A). Meanwhile, in the CIAH and the PUCHI cohort, although the ANN model did not show the best net benefit gain, the benefit was similar to that of the Eastern models and was significantly better than that of the Western models (Figure 3B,C).

**TABLE 2:** AUC of the ANN model and previous prediction models in the SEER cohort, the CIAH cohort, and the PUCHI cohort for 5‐y survival status

Model	SEER cohort		*P* value	CIAH cohort		*P* value	PUCHI cohort		*P* value
	AUC	95% CI		AUC	95% CI		AUC	95% CI	
ANN model (ref)	0.791	0.774‐0.807		0.866	0.836‐0.899		0.850	0.826‐0.874	
Western									
Kattan et al	0.788	0.771‐0.805	.512	0.828	0.796‐0.861	.001	0.821	0.794‐0.847	<.001
Kim et al	0.786	0.769‐0.803	.335	0.837	0.805‐0.869	.015	0.812	0.785‐0.838	<.001
Eastern									
Zheng et al	0.775	0.757‐0.792	<.001	0.853	0.824‐0.881	.189	0.826	0.800‐0.852	.002
Han et al	0.778	0.760‐0.795	.001	0.847	0.818‐0.876	.072	0.827	0.802‐0.853	.002
Song et al	0.754	0.736‐0.772	<.001	0.782	0.748‐0.816	<.001	0.818	0.791‐0.845	<.001
Woo et al	0.681	0.664‐0.698	<.001	0.847	0.817‐0.876	.084	0.805	0.779‐0.831	<.001
TNM 8th	0.749	0.731‐0.768	<.001	0.801	0.767‐0.835	<.001	0.821	0.795‐0.847	.001

Abbreviations: ANN, artificial neural work; AUC, area under curve; CI, confidence interval; CIAH, Cancer Institute Ariake Hospital; PUCHI, Peking University Cancer Hospital & Institute; SEER, Surveillance, Epidemiology, and End Results.

## 4 DISCUSSION

Accurate estimation of the prognosis can help in decision‐making about perioperative adjuvant treatment and follow‐up frequency, and is thus important for both patients and physicians. Although the TNM staging system is widely used, its application is limited to risk grouping. Therefore, more scoring models or nomograms for individualized survival prediction have been developed in recent years. Kattan et al developed a postoperative nomogram for disease‐specific survival (DSS) after an R0 resection for gastric cancer; the tool was widely validated with good discrimination and calibration in some high‐volume hospitals worldwide.^16^, ^41^ However, by using a cancer registry, Ashfaq et al reported that this nomogram overestimates DSS from gastric cancer in the general population.^15^ Additionally, Strong et al also found that the nomogram significantly underestimated survival in a Korean cohort.^42^ Woo et al created a novel model to predict prognosis after gastrectomy for gastric cancer; the model had a good C‐statistic of 0.798‐0.868 in four external validation cohorts in three different countries.^9^ However, the model showed a decreased C‐statistic of 0.762 when validated using the SEER dataset, but they did not show the calibration plot.^43^ A major concern in these prediction models is that they have unsatisfactory generalizability and are thus are difficult to be widely used clinically.

We speculate that race and hospital capacity are crucial for the external validation of these models; however, these two factors are often not included in most prognostic models.^12^, ^15^, ^44^ Thus, we conducted this study to develop a new comprehensive prediction model. To improve the universality of our model, we included cohorts both from the West (SEER database) and the East (CIAH cohort) into the development cohort. One advantage of the SEER database as part of the development cohort is that it contains detailed information on patient race. Moreover, the hospital capacity can also be included as a potential prognostic factor for model analysis. From the above two cohorts, 13 candidate clinicopathological variables were selected for model construction. By using a Cox regression model with LASSO penalty for reducing data dimensionality, nine factors were selected for the ANN model. Except for race and hospital capacity, the other factors are often included in other predictive models.

Consequently, we developed an ANN model for survival prediction after training. Internal and external validation of the model using three datasets from three different countries in the West and East showed it has high accuracy in predicting the 5‐year survival rate of gastric cancer patients despite the significant difference in the clinicopathologic features of the three cohorts. Our model was also superior to the 8th TNM staging system in all the three cohort. More importantly, our model can be used in patients with less than 16 lymph nodes retrieved who cannot be evaluated using the TNM staging system. While the TNM staging system is useful for risk stratification and comprises three major factors of malignancy, it cannot accurately predict prognosis. Although the ANN model comprised more factors (n = 9) than the TNM staging system and other previous prediction models, all these factors are accurately defined and are easily obtained from medical records.

We also compared the predictive power of the current model with those of previous models. In the Eastern cohorts, we found that the current model has comparable predictive capability to that of the models established in the East, while it has significantly higher predictive capability than the models established from the West.^11^, ^17^, ^38^, ^40^, ^45^ Considering that the Western data in the model development cohort accounts for a large proportion, these results strongly support the versatility of the model. In previous studies, although their ANN models showed promising results, these studies failed to provide trained models for external validation or clinical use.^27^, ^28^, ^29^, ^30^, ^46^, ^47^ In our study, the exported PMML codes are available for external validation and clinical application in other centers, Although this way is not as easy to use as the TNM staging system, it was still more convenient to use in SPSS, EXCEL, and other statistical software.

However, there are also some limitations in this study. First, both the SEER and the CIAH cohort are from open databases with limited clinical parameters. For example, information about adjuvant chemotherapy and radiotherapy are not completely available, and in the SEER cohort, there were no details on surgical resection (eg, margin status). These factors can significantly affect patient survival and we also could not compare with some models that include more detailed clinicopathologic information.^48^ Although our model showed adequate predictive capability, the lack of the above information still limits its predictive power and may explain the relatively low AUC value in the SEER cohort. Future studies should include a larger sample size and more prognostic factors to create a more robust ANN model. The prediction models combining immunoscore or radiographic signature with clinicopathologic variables by Jiang et al show future research trends and provide good examples.^49^, ^50^ Second, hospital capacity was categorized based only on estimation instead of the actual number of gastrectomy performed annually. Both CIAH and PUCHI are important gastric cancer centers where more than 200 cases of gastrectomy are conducted annually. Further, our definition of the SEER cohort as from a low‐volume center was only based on the epidemiological and therapeutic characteristics of gastric cancer in the United States. Although less than 5% of US patients were treated in a high‐volume center, our estimate lacks accuracy. Accordingly, the accuracy of our model should be further validated in high‐volume centers in the United States. In addition, the total number of retrieved lymph nodes does not necessarily reflect the quality of surgery. Finally, this was a retrospective study, and the inclusion of only patients with complete information could have introduced a selection bias.

In summary, we developed and externally validated an ANN model predicting 5‐year OS after gastrectomy for gastric cancer based on a cohort of patients from both Eastern and Western databases. Our ANN model showed significantly better discriminative capability and more accurate individualized survival prediction than the 8th AJCC TNM staging and other prediction models.

## DISCLOSURES

All the authors have no potential commercial conflict of interest.

## AUTHOR CONTRIBUTIONS

Jiafu, Ji, Xiaolong Wu, and Ziyu Li conceptualized and designed the study; Xiangji Ying and Yan Zhang involved in development of methodology and acquisition of data; Xiaolong Wu and Fei Shan analyzed and interpreted the data; Ziyu Li, Xiangyu Gao, and Xiaolong Wu drafted the article. All the authors gave the final approval of the version.

## Supporting information

Supinfo

Supinfo

## Data Availability

Data of the PUCHI cohort are not shared, owing to the privacy or ethical restrictions. Data of the SEER and CIAH cohorts are openly available through the Internet.
