# Supplementary material for: Development and validation of an artificial neural network prognostic model after gastrectomy for gastric carcinoma: An international multicenter cohort study
Source: Cancer Med. 2020 Jul 15;9(17):6205–15. doi: 10.1002/cam4.3245 (PMC7476835; doi:10.1002/cam4.3245)
Supplement: Supplementary file 1 — Supinfo [file CAM4-9-6205-s001.pdf]

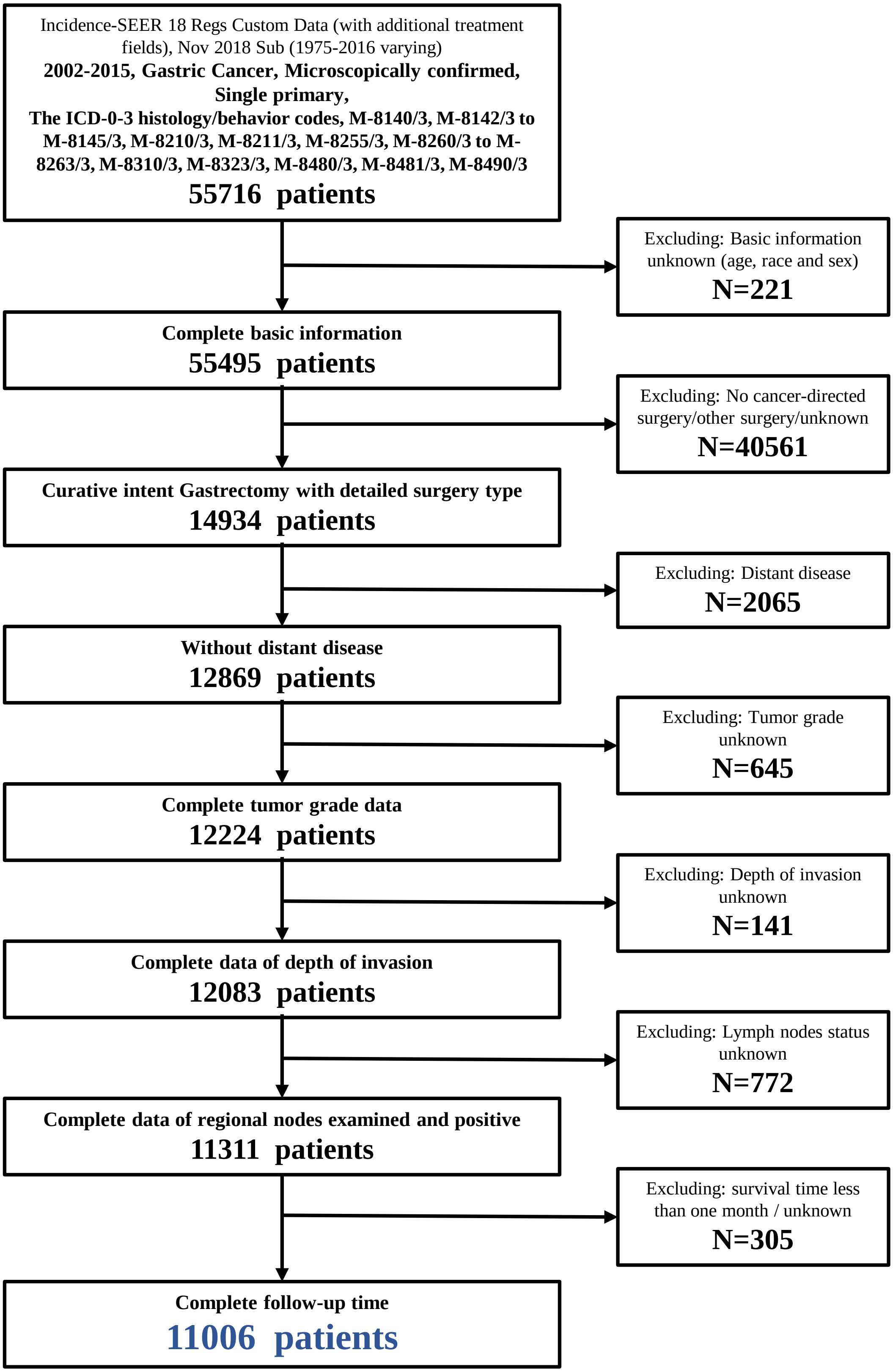

Fig. S1 Flowchart of patient selection process in the SEER cohort

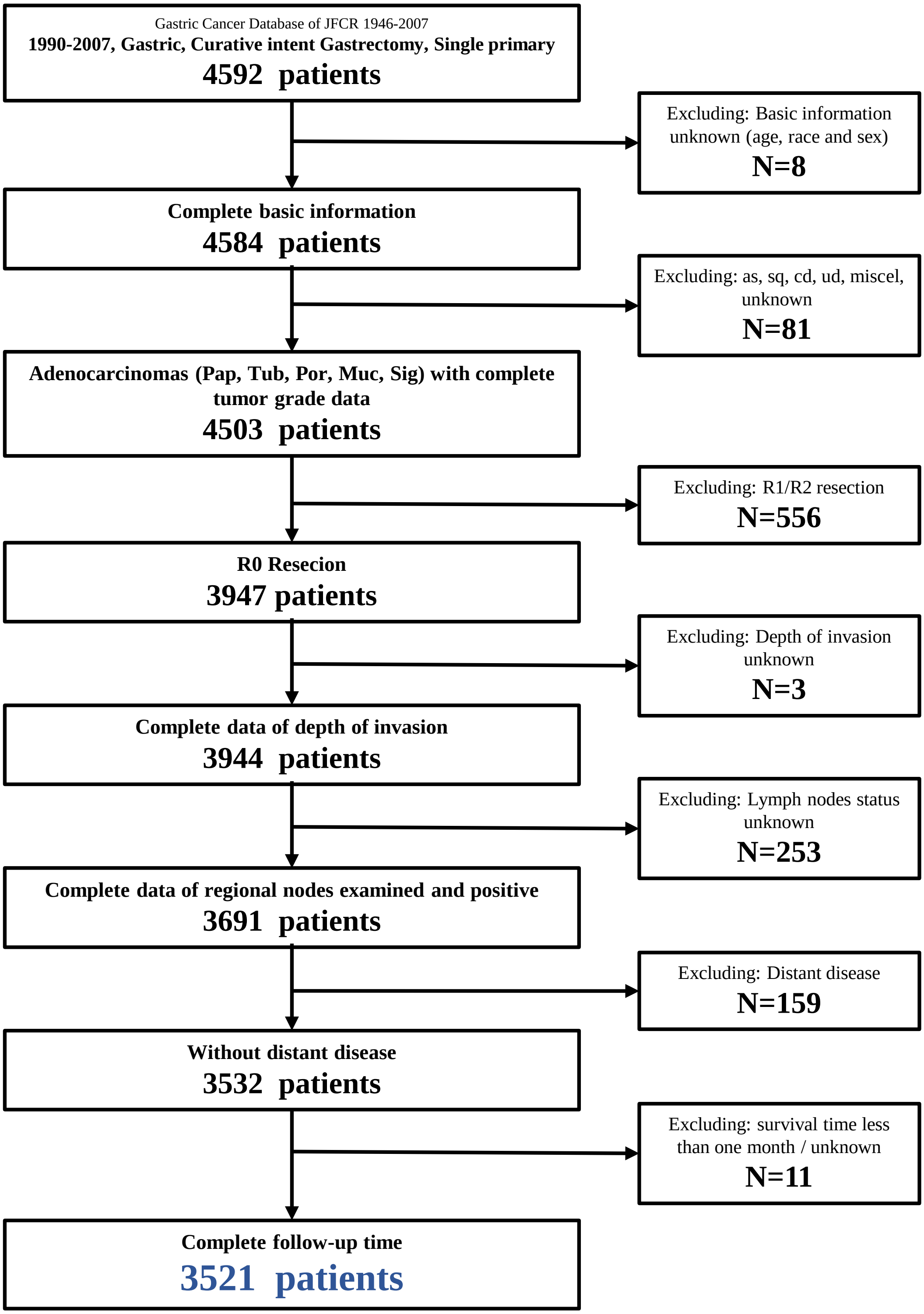

Fig. S2 Flowchart of patient selection process in the CIAH cohort

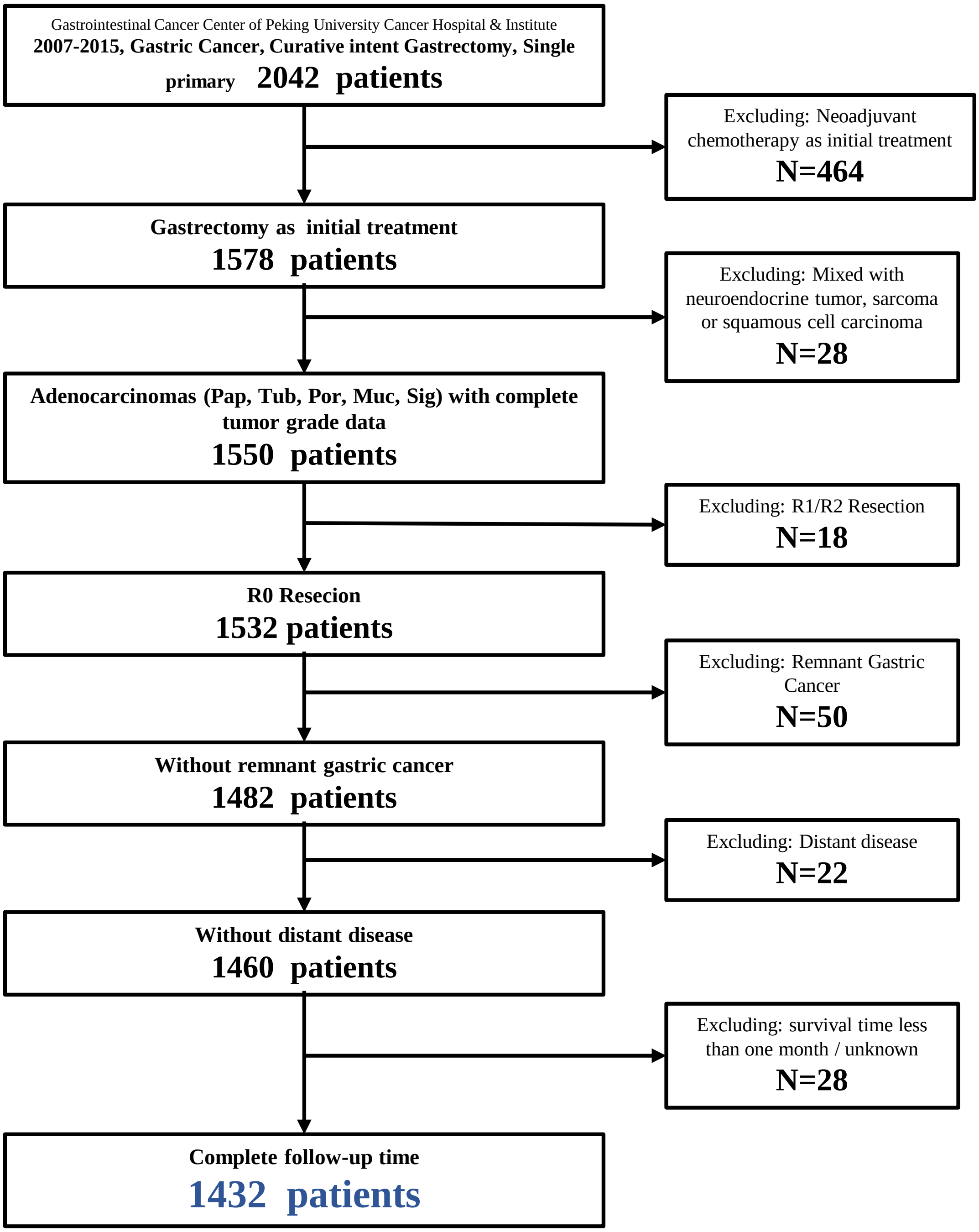

Fig. S3 Flowchart of patient selection process in the PUCHI cohort

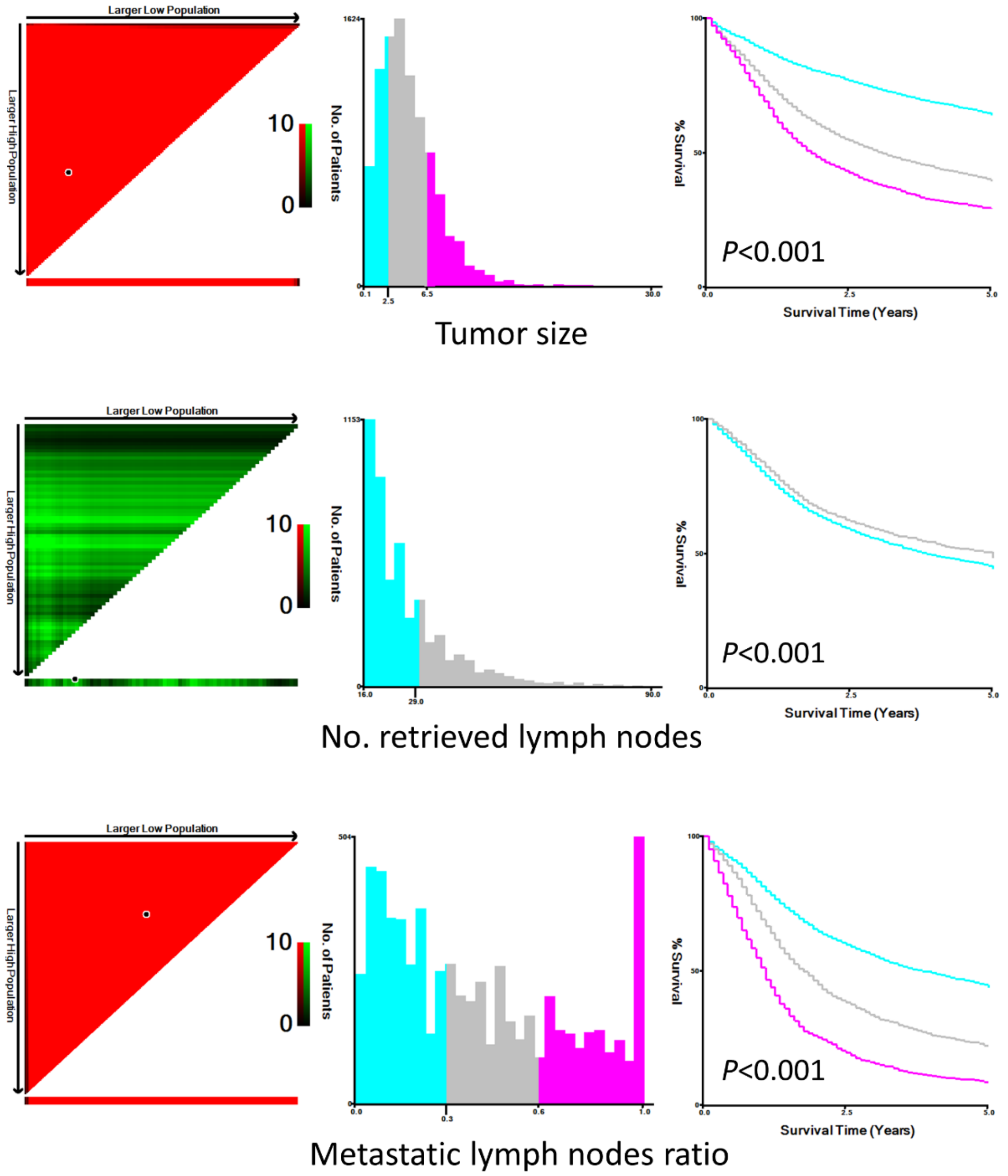

Fig. S4 X-tile analysis of the potential selected variables including tumor size, number of retrieved lymph nodes, and ratio of metastatic lymph nodes. Red coloration represent an inverse correlation with survival, whereas green coloration represents direct associations. The optimal cut-point occurs at the bright pixel (green or red).

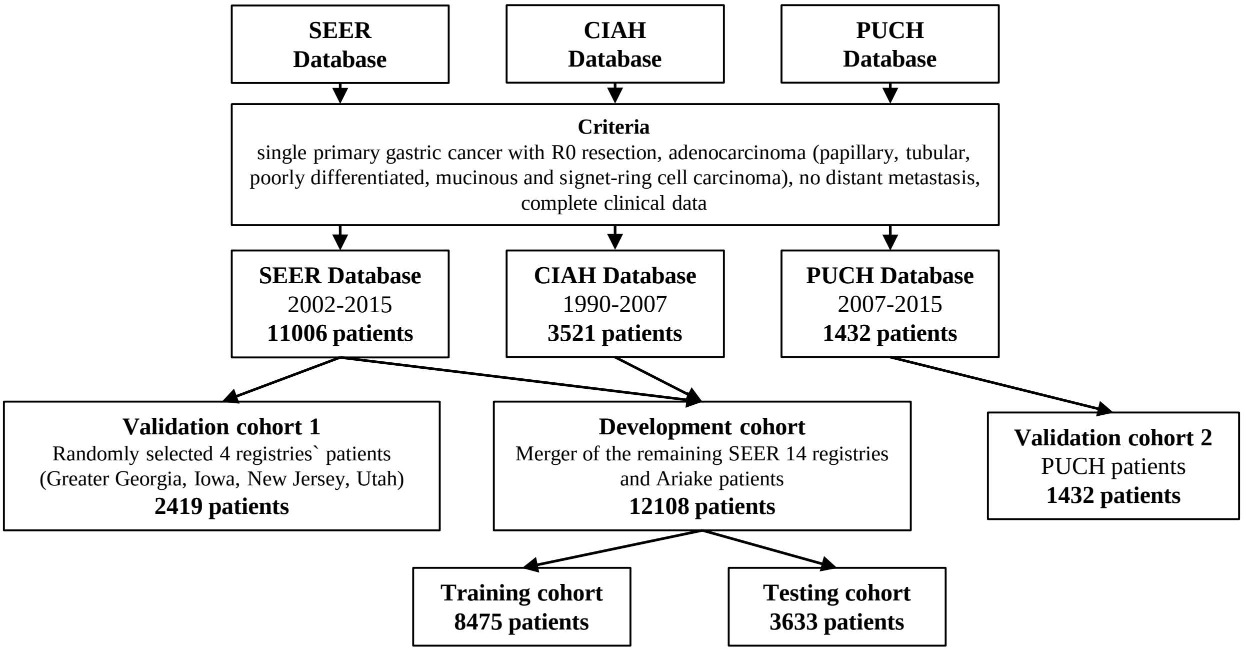

Fig. S5 Detailed grouping process for development cohort and validation cohorts

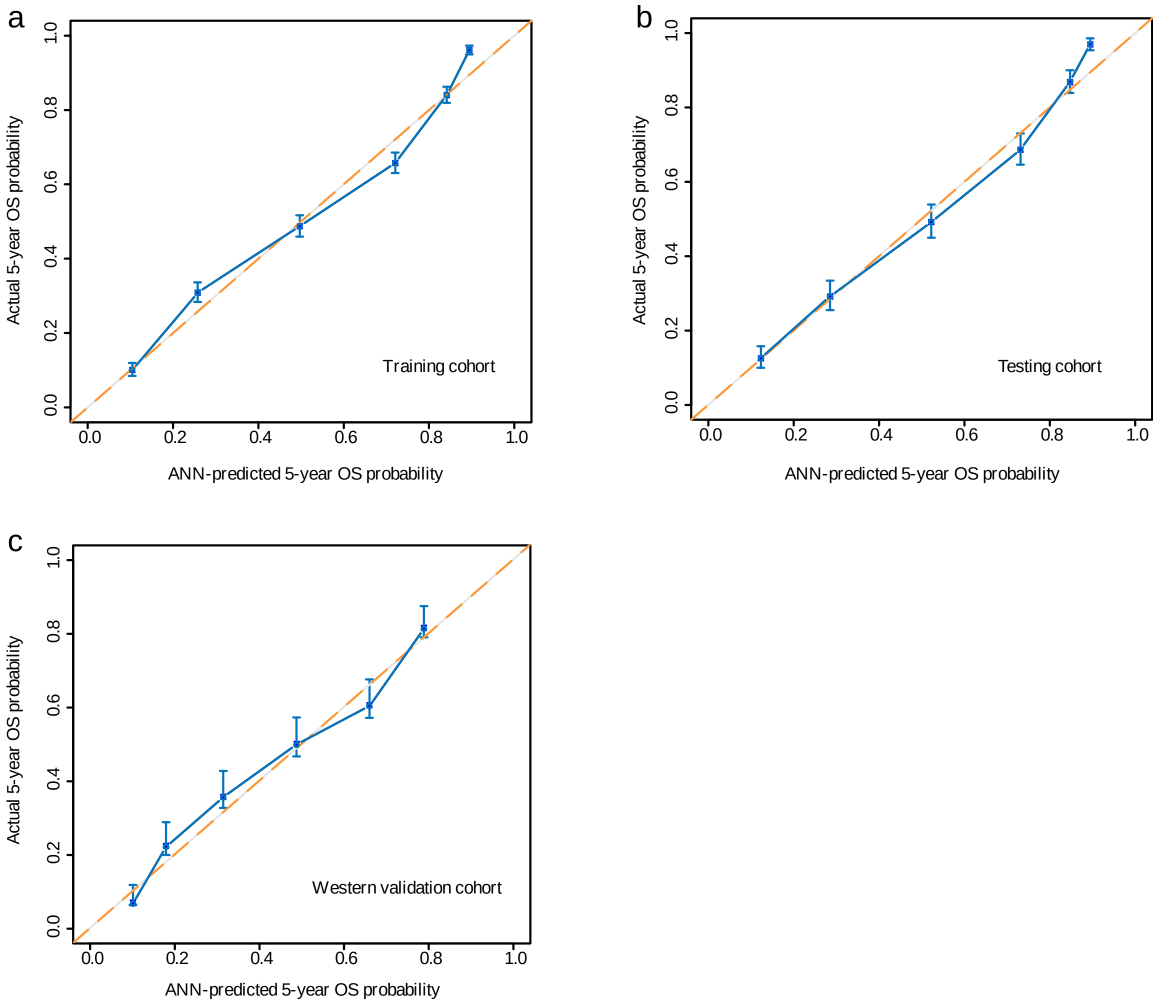

Fig. S6 Calibration of the ANN model. a. training cohort, b. testing cohort, c. Western validation cohort

a

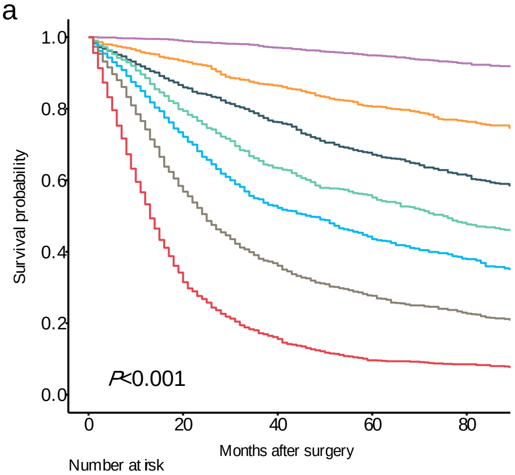

Number at risk

Group A	1778	1421	1257	1104	750
Group B	1154	902	727	556	384
Group C	1091	824	623	470	337
Group D	865	617	422	325	224
Group E	1038	687	425	297	220
Group F	1191	644	350	229	156
Group G	1358	438	178	91	69

b

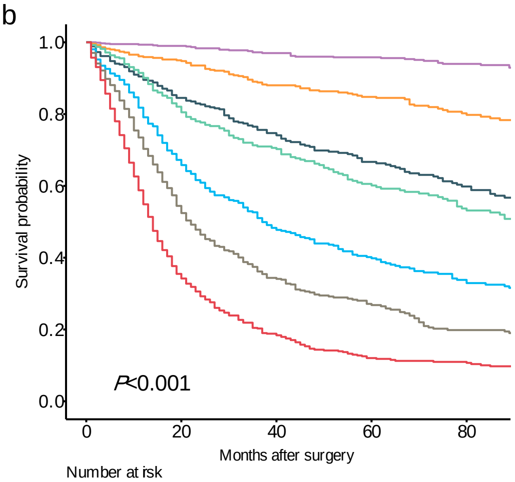

Number at risk

Group A	776	573	499	440	306
Group B	508	403	332	269	179
Group C	444	323	242	181	120
Group D	387	263	194	139	97
Group E	463	279	173	117	80
Group F	474	239	133	89	52
Group G	581	195	88	50	36

c

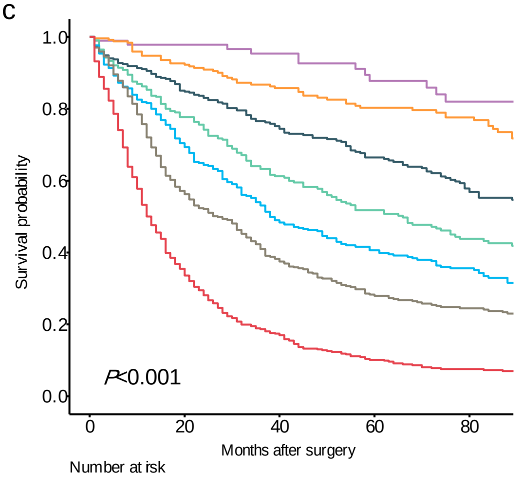

Number at risk

Group A	94	85	71	53	39
Group B	252	214	168	135	108
Group C	373	291	215	164	115
Group D	317	230	147	106	73
Group E	392	261	164	114	86
Group F	443	240	142	95	69
Group G	548	184	81	40	27

Fig. S7 Kaplan-Mier survival curves of the ANN risk subgroups. a. training cohort, b. testing cohort, c. Western validation cohort

Table S1 Demographic and clinicopathologic variables for the development cohort and the Western validation cohort

	Developing cohort (14 randomized selected registries of SEER cohort and CIAH cohort)				SEER validation cohort (Greater Georgia, New Jersey, Iowa, Utah)		*P* Value
	Training cohort		Testing cohort				
	n	%	n	%	n	%	
**Hospital volume**							<0.001
low	6040	71.3	2547	70.1	2419	100	
high	2435	28.7	1086	29.9	0		
**Age (years)**							<0.001
<41	322	3.8	125	3.4	113	4.7	
41-60	2761	32.6	1184	32.6	648	26.8	
61-70	2242	26.5	952	26.2	567	23.4	
>70	3150	37.2	1372	37.8	1091	45.1	
**Sex**							0.001
Male	5085	60	2256	62.1	1383	57.2	
Female	3390	40	1377	37.9	1036	42.8	
**Race**							<0.001
Non-Latino-White	2045	24.1	813	22.4	1330	55	
Latino-White	1364	16.1	590	16.2	311	12.9	
Black	816	9.6	343	9.4	541	22.4	
Chinese+Japanese+Korean	3597	42.4	1591	43.8	161	6.7	
Others	653	7.7	296	8.1	76	3.1	
**Tumor location**							<0.001
Upper	1251	14.8	533	14.7	329	13.6	
Middle/Lower	4972	58.7	2136	58.8	1211	50.1	
Overlapping lesion	535	6.3	243	6.7	180	7.4	
Unknown	1717	20.3	721	19.8	699	28.9	
**Tumor size (cm)**							<0.001
<2.6	2357	27.8	992	27.3	574	23.7	
2.6-6.6	4027	47.5	1743	48	1086	44.9	
>6.6	1477	17.4	654	18	394	16.3	
diffuse	151	1.8	78	2.1	35	1.4	
Unknown	463	5.5	166	4.6	330	13.6	
**Resection**							0.955
Subtotal	6611	78	2825	77.8	1884	77.9	
Total	1864	22	808	22.2	535	22.1	
**Tumor extension**							<0.001
Mucosa/Submucosa	3053	36	1279	35.2	578	23.9	
Proper muscle	1077	12.7	495	13.6	339	14	
Subserosa	2429	28.7	1067	29.4	894	37	
Serosa	1595	18.8	663	18.2	504	20.8	
Adjacent organ invasion	321	3.8	129	3.6	104	4.3	
**No. of rLN**							<0.001

1-15	3381	39.9	1448	39.9	1397	57.8	
16-29	2631	31	1140	31.4	750	31	
>29	2463	29.1	1045	28.8	272	11.2	
**No. of mLN**							<0.001
0	4316	50.9	1861	51.2	1045	43.2	
1-2	1388	16.4	615	16.9	445	18.4	
3-6	1276	15.1	541	14.9	469	19.4	
7-15	1077	12.7	454	12.5	344	14.2	
>15	418	4.9	162	4.5	116	4.8	
**MLR**							<0.001
0	4316	50.9	1861	51.2	1045	43.2	
<0.32	2223	26.2	952	26.2	646	26.7	
0.32-0.64	983	11.6	434	11.9	358	14.8	
>0.64	953	11.2	386	10.6	370	15.3	
**Differentiation**							0.050
Differentiated	3025	35.7	1304	35.9	802	35.9	
Undifferentiated	5450	64.3	2329	64.1	1617	64.1	
**Histology**							0.580
Adenocarcinoma	6349	74.9	2709	74.6	1785	73.8	
Mucinous Adenocarcinoma	180	2.1	90	2.5	52	2.1	
Signet-ring cell carcinoma	1946	23	834	23	582	24.1	

SEER Surveillance, Epidemiology, and End Results, *CIAH* Cancer Institute Ariake Hospital, *rLN* retrieved lymph nodes, *mLN* metastatic lymph nodes, *MLR* metastatic lymph nodes ratio

Table S2 ANN variable corresponding codes

Variables	Code	Value
Hospital_volume	0	low
	1	high
Age	1	<41
	2	41-60
	3	61-70
	4	>70
Race	1	Non-Latino-White
	2	Latino-White
	3	Black
	4	Chinese+Japanese+Korea
	5	Others
Gastrectomy_type	0	subtotal
	1	total
Tumor_size	1	<2.6
	2	2.6-6.6
	3	>6.6
	4	diffuse
	5	Unknown
Depth_of_invasion	1	Mucosa and Submucosa
	2	Proper muscle
	3	Subserosa
	4	Serosa
	5	Adjacent organ invasion
No.metastatic_LN	0	0
	1	1-2
	2	3-6
	3	7-15
	4	>15
No.retrieved_LN	1	1-15
	2	16-29
	3	≥30
metastatic_LN_ratio	0	0
	1	<0.32
	2	0.32-0.64
	3	≥0.64
